# From Structure Prediction to Genomic Screens for Novel Non-Coding RNAs

**DOI:** 10.1371/journal.pcbi.1002100

**Published:** 2011-08-04

**Authors:** Jan Gorodkin, Ivo L. Hofacker

**Affiliations:** 1 Center for non-coding RNA in Technology and Health, IBHV University of Copenhagen, Frederiksberg, Denmark; 2 Division of Genetics and Bioinformatics, IBHV University of Copenhagen, Frederiksberg, Denmark; 3 Department of Theoretical Chemistry, University of Vienna, Wien, Austria; Stanford University, United States of America

## Abstract

Non-coding RNAs (ncRNAs) are receiving more and more attention not only as an abundant class of genes, but also as regulatory structural elements (some located in mRNAs). A key feature of RNA function is its structure. Computational methods were developed early for folding and prediction of RNA structure with the aim of assisting in functional analysis. With the discovery of more and more ncRNAs, it has become clear that a large fraction of these are highly structured. Interestingly, a large part of the structure is comprised of regular Watson-Crick and GU wobble base pairs. This and the increased amount of available genomes have made it possible to employ structure-based methods for genomic screens. The field has moved from folding prediction of single sequences to computational screens for ncRNAs in genomic sequence using the RNA structure as the main characteristic feature. Whereas early methods focused on energy-directed folding of single sequences, comparative analysis based on structure preserving changes of base pairs has been efficient in improving accuracy, and today this constitutes a key component in genomic screens. Here, we cover the basic principles of RNA folding and touch upon some of the concepts in current methods that have been applied in genomic screens for *de novo* RNA structures in searches for novel ncRNA genes and regulatory RNA structure on mRNAs. We discuss the strengths and weaknesses of the different strategies and how they can complement each other.

## Introduction

Non-coding RNA genes (ncRNAs) have emerged as major players in the cell and are involved in both housekeeping functions as well as regulation. They are characterized as functional transcripts that do not code for proteins and can be processed in numerous ways, see e.g., [1], [2]. An abundant class of ncRNA genes are the micro RNAs (miRNAs), which have received considerable attention e.g., [3]–[5]. This can be observed through the rapid growth in the literature, not only for miRNAs [6], but also for ncRNAs in general [7]. Furthermore, regulatory RNA structure in UTR regions of protein-coding genes is also an exciting, emerging field.

The roles of ncRNAs are diverse and not only include regulation of protein coding genes [8], but also inactivation of other gene classes (e.g., imprinting [9], [10]), alternative splicing [11], and modifying other ncRNAs [12], to mention just a few examples. Thus the miRNAs are but one among several other classes of ncRNAs. Novel classes of small ncRNA genes such as piRNAs [13], [14] and hpRNAs [15] have also been reported. Recently, long intervening ncRNAs (lincRNAs) have been found. These are mRNA-like transcripts that lack protein-coding potential, contain exon intron structure, and are apparently largely unstructured [16]. The repertoire of ncRNAs is rapidly expanding and RNA-seq sequencing techniques, in combination with computer methods, are expected to give rise to a general expansion of the RNA universe. These RNA families are collected in the Rfam database [17] in the form of structural alignments and consensus structures. In a number of cases, such as SRP RNAs and tmRNAs [18], Rfam is based on pre-existing curated RNA structural alignments from specialized databases. This important resource is also often used to construct and test RNA structure prediction tools [7].

The size variation of ncRNAs is extreme, ranging from 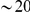 nucleotides (nt) for small interfering RNAs and miRNAs to 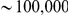 nt for the *air* RNA [10]. ncRNAs are not only located in intergenic regions, that is outside of protein coding genic regions, but they are also found in introns. In the latter case they are either processed out during splicing, or they represent independent transcripts that come with their own promoters, as e.g., in *Caenorhabditis elegans* [19]. There are also examples of ncRNAs overlapping coding regions [20]. In addition, mRNAs may contain functional *cis*-acting RNA structures, such as the iron-responsive element [21] in vertebrates or riboswitches in bacteria [22].

As can be seen by inspection of Rfam, a solid volume of ncRNAs and regulatory RNAs come with a characteristic and functional RNA structure, which often is more conserved in evolution than its primary sequence. In order to find ncRNA genes, it therefore makes sense to search for RNA (secondary) structure rather than primary sequence. Computationally, this is a much more challenging and demanding problem than searching protein coding space, as there are no regular signals in RNA structured sequence such as open reading frames.

However, searching for RNA secondary structure is likely not to provide us with all functional non-protein-coding transcripts, since the emerging compilation of long ncRNAs seems to indicate that these in general are not densely structured, even though they might contain structural regions. This is exemplified by a mouse transcriptomic analysis that revealed thousands of such transcripts based upon full-length cDNA sequencing [23]. It remains to be systematically investigated whether these RNAs harbor characteristic structures that carry out specific functions, and thus if searching for RNA structure in general is a sufficient starting point to search for ncRNAs. It is worth noting that the only functionally well-characterized lncRNA, hotair, does have functional RNA structures 24–26.

Here, we focus on describing the principles of searching for *de novo* RNA structures in genomic sequences, being aware that the gene (and functional transcript) itself can be (much) larger than the (predicted) structure and that overlapping predicted structures can be in the same functional transcript.

## Parameters of the Search Space

Searching for novel RNA secondary structures requires that functional structures can be distinguished (e.g., by their folding energy) from those generated on shuffled sequences of the same composition, that is, the background. In general, programs like mfold and RNAfold will fold *any* RNA sequence you feed into them. Whether the structure (or parts thereof) is actually trustworthy is of course determined by the user.

It turns out that for most known ncRNAs (with miRNAs as a notable exception), it is generally not sufficient to screen individual genomes using minimum free energy folding (of a sequence in some fixed size window), since neither folding energies nor the resulting structures provide a reliable signal. Although ncRNAs tend to have somewhat more stable structures than expected by chance, the difference in folding energies between random sequences created by shuffling and native ncRNAs is in general too small to distinguish real ncRNAs from decoys [27], [28]. However, the often stronger conservation of the (secondary) structure compared to the primary sequence can be used to enhance the discrepancy to the background. For example, for a human RNA sequence CCCCCCCAGUUGGGGGG that forms a simple hairpin, the mouse version could be CACCCCCAGUUGGGGUG such that a GC base pair in human corresponds to an AU base pair in mouse. Not only do such features destroy conservation of primary sequence, but the base pairs can also be separated essentially by the full length of the sequence. Hence, meaningful *in silico* screens can be carried out on comparative genomic data, but using complex algorithms that take long-range base pairs into account.

**Figure 1:**
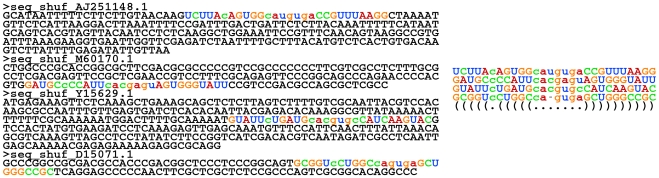
Searching for common RNA secondary structure in unaligned sequences. The scenario of searching for common RNA structure in sequences (left) that are otherwise unrelated (here generated by shuffling the order of the nucleotides in real sequences). This structure can either represent portions of an ncRNA gene or a structural RNA element in an mRNA. The search result in a multiple structural alignment (right) is typically based on the pattern of obtained compensating changes.

The ideal search scenario is illustrated by a toy example in Figure 1, where we have randomized some sequences (shuffling the order of the nucleotides) and implanted a small hairpin conserved only in structure. These can be considered as a set of corresponding (but poorly conserved) sequences that do not necessarily have much in common except for common RNA structure. This sequence set can be searched (sequences on the left side) and a joint structure extracted (on the right side) where base pairs are represented by matching parentheses. In real examples, the “background part” of sequences is never so strongly divergent and neither are the sequences of the contained motifs (while the structure is convergent). This, of course, creates challenges for the prediction scheme.

Overall, *in silico* searches for ncRNAs can in essence be carried out in the following three ways: (i) by sequence/structure similarity to already known ncRNAs, (ii) by searching for specific ncRNA classes, e.g., miRNAs and snoRNAs, and (iii) *de novo* searches. Here, we focus on *de novo* searches, but briefly touch upon the others below.

## Sequence Similarity Search for ncRNAs

The basic form of similarity search is purely sequence based using BLAST [29], and this approach has apparently not been reported in the literature for anything other than finding near identical sequences, e.g., genome and EST annotation projects [30], [31]. The more advanced approach is to include the RNA secondary structure as done for covariance models such as INFERNAL and RaveNnA [32]–[34]. These constitute a probabilistic model of the RNA structure together with the corresponding sequence variation (e.g., compensating base pairs). More specifically, they employ *stochastic context-free grammars* (SCFGs), an extension of hidden Markov models (HMMs), that can cope with the long-range base pair interactions. An alternative (which is faster) is to extract patterns for RNA motif search, e.g., RNAmotif [35].

To obtain good models, well-curated data (structural RNA alignments) are needed, which can be obtained either from specialized databases, as in the case of RNAseP RNA and SRP RNAs [18], [36], or from the meta database Rfam. Curating these and conducting homology-based searches comes with its own set of issues, which is described elsewhere [7].

Class-specific searches use distinctive features of an RNA class to search for novel, but not necessarily homologous, members of that class. miRNAs are such an example that can be identified on the basis of the characteristic stem-loop shape of the precursor either encoded as explicit rules or combined with machine learning techniques [6]. Another example is the well-known tRNA-scanSE program to search for tRNAs [37]. Similar types of searches have also been employed for other families, and incorporating this information is generally expected to help span greater distances in the evolutionary tree than what can be done solely from (present) covariance models. The principal reason is RNA structure itself changes, so that models made for one family cannot readily be applied to another. Well-known examples are RNAse P RNA [12] and telomerase RNA [38]. A recent advance in the INFERNAL package is that it can search for local structural matches.

As previously mentioned, *in silico* screens currently involve searching for *de novo* RNA structure, but there have also been a few cases employing GC content as an indicator of RNA sequence structure in certain organisms (extremophiles with biased AU content) [39]–[41]. Here, we focus on describing the principles and the concepts of *de novo* searches. When there is overlap with similarity search methods, this will be mentioned. We will concentrate, however, on the concepts and not on reporting what one actually can expect to find and what to do with these sequences. The latter aspects are reviewed e.g., in [42].

**Figure 2:**
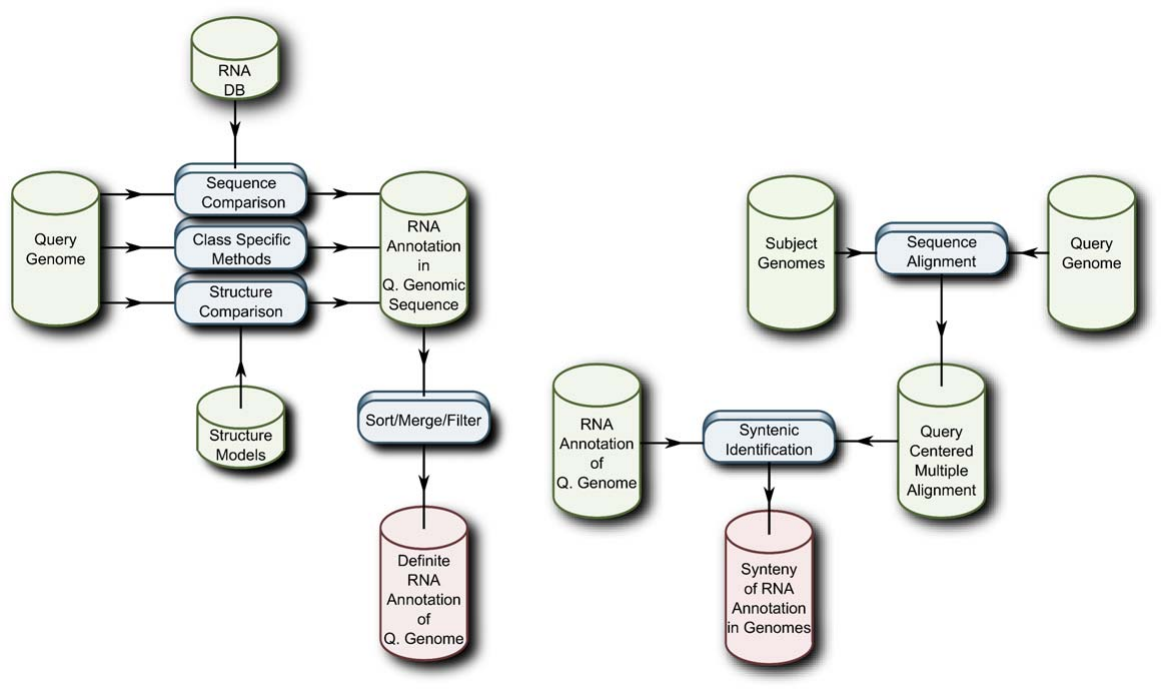
Basic flow homology (left) search in combination with identification of syntenic regions (right) of related genomes. (Figure courtesy of Christian Anthon.)

To summarize, in Figure 2 there are two basic flow charts of current similarity searches to provide mapping of homologous ncRNAs and regions of synteny for related genomes. The latter can be used as an extra layer of confirmation for the raw similarity search, but also to investigate if genomic rearrangements have taken place. Clearly, synteny can yield further support for the outcome of an *in silico* screen.

## RNA Structure and Folding

**Figure 3:**
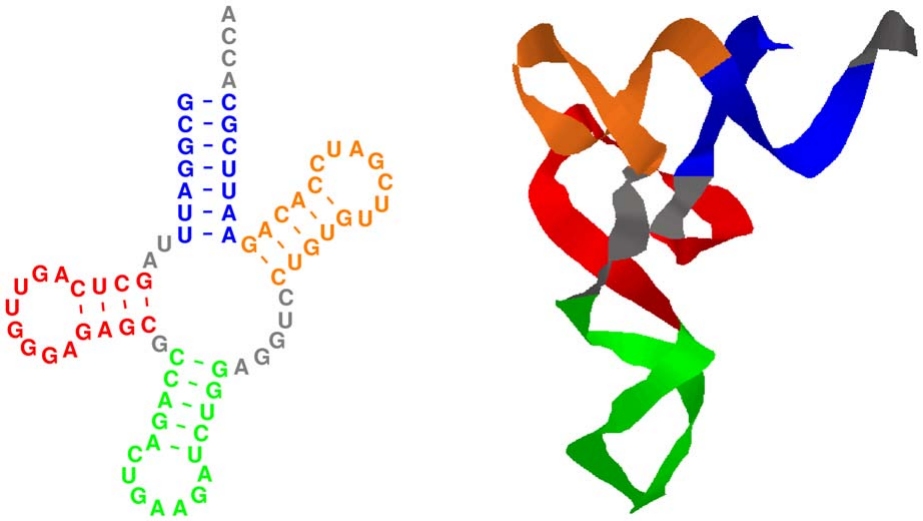
An example of 2D (left) and 3D (right) representations of RNA structures, here illustrated for a tRNA. The RNA secondary structure is an important step towards the full 3D structure. (Figure from [116].)

**Figure 4:**
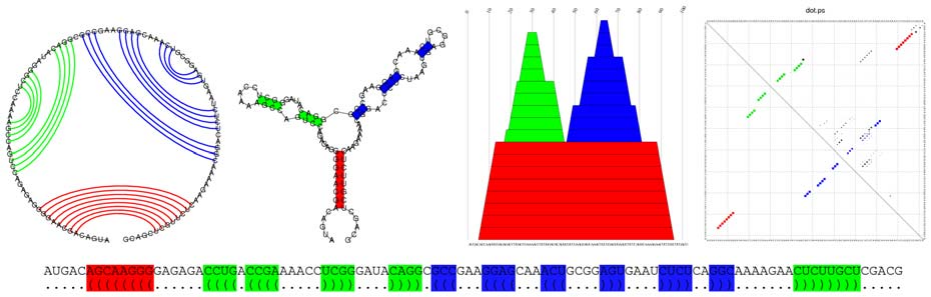
Representations of RNA (secondary) structure. From top left: A circle plot, a conventional secondary structure diagram, a mountain plot, and a dot plot. The bottom diagram shows the secondary structure in dot-bracket notation, where a base pair is represented by matching parentheses. The respective colors in each diagram represent the same base pairs. The structure shown is a glycine riboswitch from *B. subtilis*, Rfam family RF00504.

As mentioned above, folding of single sequences is in general not sufficient to reliably detect RNA structure. Still, the principle of folding single sequences is fundamental in basically all computational approaches constructed to search for RNA structure in genomic sequence. The structured RNA molecules by nature take a characteristic three-dimensional (3D) structure. As depicted in Figure 3, even though it is still difficult to predict 3D from 2D structure, most contacts between bases are already part of the secondary structure. Moreover, the canonical base pairs making up the secondary structure can be reasonably well predicted without any knowledge of tertiary structure. This makes the minimum free energy secondary structure a useful abstraction of the full 3D structure. Current methods do generally focus on the RNA secondary structure, even though the awareness and feasibility of taking the 3D structure into account is improving. It is beyond the scope of this text to go deeper into this. Unless mentioned otherwise, we will from now on write RNA structure as a shorthand for RNA secondary structure. The RNA secondary structure can be represented in numerous ways, as depicted in Figure 4.

### Concepts of Folding RNA Sequences

The basic folding algorithm goes back to the early work of Ruth Nussinov [43], who proposed a simple dynamic programming algorithm to find the maximum number of base pairs for an RNA sequence. The idea is to keep track of the number of base pairs of any sub-sequence starting at some position, say 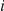, and ending at position 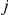. Given that the sequence is 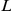 nucleotides long, the recursion requires that 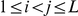. Additionally, pseudoknots are ignored as a first approximation. Pseudoknots can be considered as higher-order base pairing interactions and would correspond to having lines crossing in the outer left part of diagram shown in Figure 4. Including pseudoknots results in much more complex algorithms with higher time and memory consumption.

**Figure 5:**
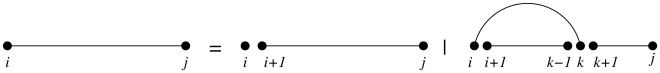
Decomposition of RNA secondary structures for the Nussinov algorithm. The decomposition is unambiguous in the sense that each structure can only be decomposed in a single way.

Thus, starting with (unpaired) sub-sequences of length one and extending (and meeting the first base pair at some point), one can consider a structure on the sub-sequence 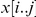. Such structure can be formed in only two distinct ways from shorter structures: Either the starting nucleotide 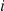 is unpaired, in which case it is followed by an arbitrary structure on the shorter sequence 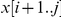, or the first nucleotide is paired with some partner base, say 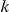. In the latter case the rule that base pairs must not cross implies that we have independent secondary structures on the sub-intervals 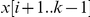 and 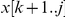. Graphically, we can write this decomposition of the set of structures as shown in Figure 5.

**Figure 6:**
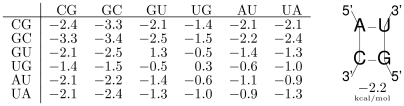
Free energies for stacked pairs and loops in kcal/mol. Note that both base pairs have to be read in 5′-3′ direction.

Denoting 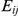 as the maximum number of base pairs (or optimal energy) for a secondary structure on 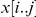 corresponding to the left side of the equation, we see that 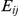 is the optimal choice among each of the alternatives. In this context, independence of two substructures in the paired cases implies that we have to optimize these substructures independently. Using 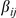 as 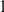 if 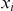 and 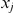 base pair and zero otherwise, we arrive at the recursion:
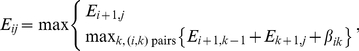
(1)where the maximum runs over 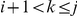. Rather than having the parameter 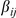 one or zero and rather than counting the maximum number of base pairs, we can let 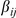 take negative values depending on the type of base pair, that is, by replacing 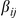 with 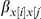 to take the individual base pairs into account, and then replace the 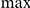 in the recursion by 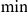. An example of filling out the dynamical programming matrix is shown in Figure 6. The recursion in Equation 1 is a simplification (and less ambiguous) of a more general form of the Nussinov algorithm. A good introduction is given in [44].

### Towards a Full Folding Algorithm

**Figure 7:**
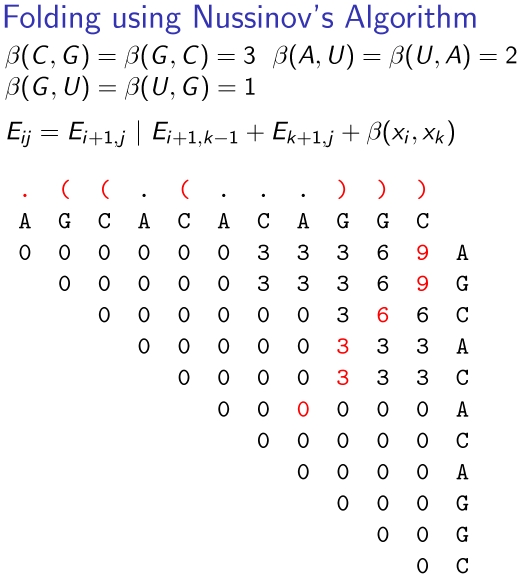
Filled dynamic programming matrix 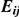 for the toy sequence AGCACACAGGC. Values giving rise to the optimal folding energy of 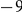 are shown in red.

This simple model is still too inaccurate, since it does not capture energetically important structure motifs, such as stacked pairs, bulges, and various types of loops (hairpin, multi, interior, and exterior). The more realistic “nearest-neighbor” energy model is therefore based on loops, rather than base pairs. A complete set of loop energies is available from the group of Doug Turner [45]. Stacked pairs, for example, consist of two consecutive base pairs and are the major source of stabilizing energy. Each possible stacking comes with its own free energy as listed in Figure 7. It can be observed that GCs have lower binding values and therefore form more stable stacks and thereby structures. This relates to the issue of searching for RNA structures in GC-rich regions in the genomes. In general, loop energies depend on the loop type and its size, and sequence dependence is conferred only through the base pairs closing the loop and the unpaired bases directly adjacent to the pair (the *terminal mismatches*). The general form of loop energy is therefore
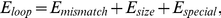
(2)where the last term is used for special cases, e.g., to assign bonus energies to unusually stable *tetra loops*. While the model allows only Watson-Crick (AU, UA, CG, and GC) and wobble pairs (GU, UG), non-standard base pairs in helices are treated as special types of interior loops. Therefore, an extended dynamic programming algorithm is needed and replaces the one shown above.

Using the loop-based energy model is essential in order to achieve reasonable prediction accuracies. On average, current energy models achieve accuracies of 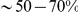 in terms of the percentage of correct base pairs [46]. Prediction accuracy tends to fall somewhat with sequence length [47]. This effect could be simply due to combinatorics (long RNAs have more *wrong* structures), or because long sequences are kinetically trapped in structures other than the ground state. Recent approaches combine structure-probing experiments and use the following information for single/double-stranded positions as constraints to the folding algorithms to obtain higher accuracy [48], [49].

The more standard energy model results in somewhat more complicated recursions and requires additional tables. However, memory and CPU requirements remain 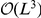 and 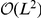 as in the Nussinov algorithm. The factor 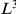 comes from the time it takes to fill out the upper half of the matrix of size 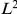 and then check for adding sub-structures (the 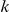 index in Equation 1). The crucial quantity in the loop-based version is the optimal free energy for a sub-sequence 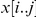 enclosed by a base pair 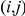. In order to compute that, we now have to distinguish between the different types of loops that can be closed by 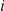 and 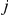. For a complete set of corresponding recursions see e.g., [50].

### Folding of Randomized Sequences

**Figure 8:**
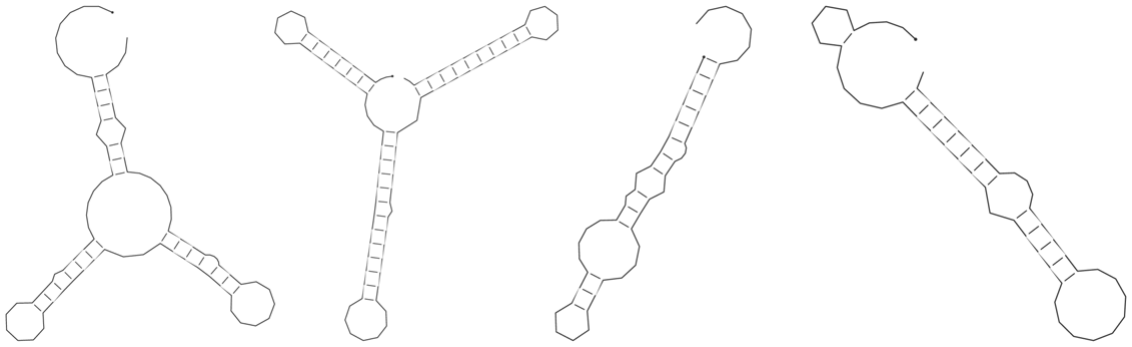
Structure prediction for two non-coding RNA sequences (DsrA and DicF) and respective (shuffled) sequences with the same length and nucleotide composition. Most readers will not be able to distinguish between the real and randomized scenarios.

While it seems natural to detect ncRNA genes on the basis of structure prediction, the task is far from straightforward. The problem is that almost any RNA sequence will form some kind of secondary structure. The real challenge is therefore to distinguish whether a structure is spurious or may constitute a functional structure. Unfortunately, structures formed by functional ncRNAs do not look significantly different from structures formed by random sequences [51], as illustrated in Figure 8. By random sequences we denote sequences for which the order of the nucleotides has been shuffled. Often this is done by preserving the di-nucleotide order, as that has an impact on the stacking of base pairs.

In fact, when Rivas and Eddy set out to build a general RNA gene finder based on this principle, they had to conclude that secondary structure alone is generally not significant enough for the detection of ncRNAs [27]. Subsequent studies [52] focused on folding energies and showed that (i) functional RNAs tend to be slightly more stable than randomized RNAs, (ii) the difference is statistically significant, but too small to be of much use without additional criteria, and (iii) that for a fair comparison randomized sequences should be generated such that the di-nucleotide content (not just nucleotide composition) is conserved.

A notable exception are microRNAs [53] which form unusually stable structures.

## Extracting Structure from Multiple RNA Sequences

As single sequences are not sufficient to extract a clear signature of RNA structure, and since RNA structure can be more conserved than sequence, multiple (orthologous/syntenic) sequences can be searched to find a common structure. It is particularly of interest to detect or exploit *compensating* base changes, as these indicate conserved structure in spite of varying sequence as exemplified in the toy example in Figure 1. Below, we conceptually describe approaches to predicting consensus RNA structure from either aligned or unaligned sequences, an essential step towards searching for RNA structure in genomic sequence.

### Mutual Information

Given a multiple sequence alignment (typically made without knowledge of the structure), the most common way to quantify covariation for the purpose of RNA secondary determination is by measuring the *mutual information* content [54], [55]:
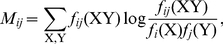
(3)where 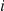 and 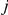 are two columns of a multiple sequence alignment, 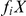 denotes the frequency of nucleotide 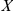 in column 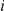, and 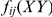 denotes the frequency of co-occurrence of the nucleotides 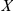 and 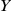.

Mutual information makes no use of pairing rules and can therefore be used to detect tertiary interactions as well. However, the number of sequences needed to reliably deduce secondary structures from mutual information alone is prohibitive for most classes of RNA. Nonetheless, alternative versions of the mutual information content have been shown to drastically lower the required number of sequences [56]–[58]. In any case, however, it makes good sense to combine co-variance analysis with structure prediction techniques. A manual approach to optimize the alignment is to revise the alignment based upon computation of the mutual information content, a process which recently has been automated in several projects, e.g., [59]–[61]. In a prediction screen, the consensus structure predictions are often based on a fixed pre-computed sequence alignment.

### Folding Multiple Alignments of RNA Sequences

Consider a multiple alignment for which the mutual information content has been computed, then one simple way to extract the information about base pairs would be to employ a Nussinov-style algorithm to maximize the amount of mutual information between paired columns. In general, such an approach is insufficient, as a number of structural features cannot be taken into account, for example base pair stacking. An alternative is to combine the information from covarying base changes with a standard dynamic programming folding algorithm. In the RNAalifold program this is done simply by averaging the folding energy over all sequences, thus, e.g., the energy contribution of a stacked pair in the consensus structure is taken as the average of the stacking energy over all sequences in the alignment. To make best use of the covariation information, this average folding energy is augmented by a covariance term that is added as a pseduo-energy. Instead of mutual information (Equation 3), the following covariation term is employed:
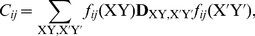
(4)where the 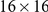 matrix 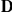 is chosen such that compensatory mutations receive a bonus of 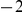 kcal/mol, consistent mutations (such as G-C 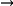 C-U) receive 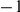 kcal/mol, conserved pairs get a score of 0, and non-canonical pairs incur a penalty of 1 kcal/mol. In contrast to mutual information, this covariance term explicitly favors consistent mutation and tends to be less noisy for alignments with few sequences.

A widely used alternative, but similar approach, is to compute probabilities for alignment columns (based on 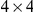 substitution rates) to be single stranded (unpaired) and probabilities for columns to be base paired (based on 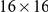 substitution rates) and search for the structure that leads to the highest alignment probability. This approach is taken in the SCFG program Pfold, which aims to maximize the joint probability of consensus structure and alignment [62]. More precisely, it computes the probability 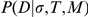 of an alignment 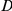 given a consensus structure 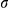, a phylogenetic tree 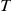, and a model of substitution rates 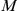. This uses a Felsenstein model [63], as is usual in maximum likelihood tree estimation, for single-stranded and base-paired columns, respectively. In addition, it uses an SCFG to compute the prior probability of a structure 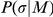, and thereby the joint probability 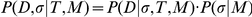. Recently, the concepts of Pfold were extended to a maximum expected accuracy framework, PETfold, to simultaneously optimize phylogenetic and energetic information [64].

Under ideal conditions, i.e., well-conserved structure, many compensatory mutations, and error-free alignments, all these algorithms produce near-perfect predictions. For realistic datasets, the challenges lie in dealing with (small) structural variations between the sequences, while being not too sensitive to alignment errors, and dealing gracefully with the lack of covariation.

### Simultaneously Folding and Aligning RNA Sequences

**Figure 9:**
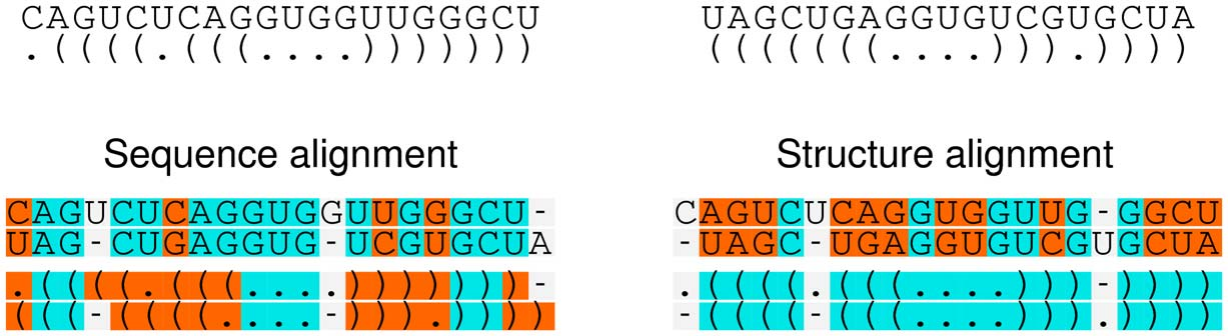
Two toy sequences that, if aligned only by their sequence, do not match in secondary structure. If correctly aligned, low sequence similarity between the two sequences does not hinder the revelation of structure.

Consensus structure prediction exploits the co-variation signal in an alignment, and this signal should increase as sequences become more diverged. A potential problem in applying sequence-based alignments for RNA structure prediction is, however, that with lower sequence similarity, alignments become more inaccurate, eventually leading to a breakdown of structure prediction. Empirically, this limit has been found to lie at about 60% pairwise sequence identity, both for RNAalifoldZ [65] and in a study by Gardner et al. [66], who showed for tRNAs that around this similarity sequence-based alignment methods drastically lose the ability to reproduce the alignment, whereas structure-based methods are still providing fairly good results. A toy example in Figure 9 illustrates how sequence similarity can be insufficient for comparing structured RNA sequences.

In 1985, Sankoff [67] published the first method for simultaneously folding and aligning 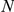 sequences of length 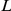, a method that has time and memory complexities of 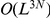 and 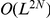, respectively. This basically makes the algorithm intractable for more than two sequences as well as for long sequences. Intuitively, for two sequences all folds in the one sequence are to be compared with all folds in the other, leading to twice as high an exponent, e.g., 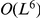 instead of 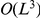. This intractable high complexity has prompted several creative attempts at simplified versions of the Sankoff algorithm, as well as completely different types of approaches, e.g., [68]. Complementary to folding alignments, approaches folding the individual sequences and aligning the structures have been proposed, e.g., [69].

Some of the first implementations for RNA structure alignments are based on SCFGs [70], [71] and avoid the high cost of the Sankoff algorithm by using an iterative approach that alternates between aligning sequences to a covariance model and deducing a refined covariance model from the alignment ([70]).

The first simplified implementation of the Sankoff algorithm was the first version of FOLDALIGN [72], which was restricted to stem-loop structures only. Later, more complete versions were published and the first full-scale implementation for two sequences was dynalign [73], [74]. A nice SCFG framework was also introduced in stemloc and later consan methods [75]–[77]. Later, PMcomp [78] and LocARNA [79] introduced the use of pre-computed base pair probability matrices to reduce computational cost (PMcomp) and memory (LocARNA). Common for these methods is that when structurally aligning two sequences, the recursion involves a *four dimensional* dynamical programming matrix. Essentially, Equation 1 can be extended to a 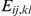 where the sub-sequences 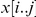 and 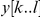 are simultaneously folded and aligned. The scoring scheme (energy model) thus has to be able to score (mis)matches between unpaired nucleotides as well as between base pairs. For the latter, one often uses the so-called ribosum matrices [80], derived from substitution frequencies in ribosomal RNAs, but also pair probabilities or even the energies of base pair stacking.

**Figure 10:**
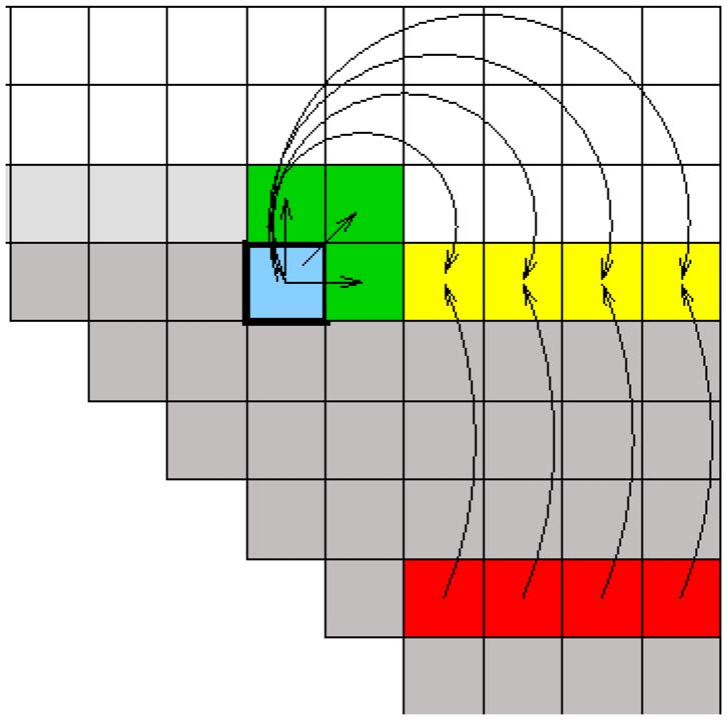
Filling out the dynamical programming matrix “ahead of time”. That is, for the current position in the sequence just partially filling out future cells, either for the first time, or by updating the maximum score in the particular cell. All grey cells, including the blue cell and the current cell (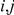 of a single sequence), have been completely computed. The green and yellow cells are partially filled out, making part use of the red cells (previously computed). (The figure is from the supplemental material of [81].)

Recently, basic conceptual improvements to the Sankoff-style approach as introduced in FOLDALIGN [81] have been implemented. The first improvement was introduction of *sparsification*, in which not all computations of what correspond to the 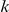 index in the Equation 1 need to be carried out, as a number of configurations are the same, but obtained in different ways from composition of various sub-structures. The other improvement was a heuristic approach that basically *prunes* away cells in the dynamical programming matrix that never exceed a length-dependent threshold. This could be accomplished by filling out the dynamical programming matrix “ahead of time” (see Figure 10 for details).

Additional methods (not explicitly employed for ncRNA gene finding) have been published since and we refer to [42] for further details.

Whereas most methods perform *global* alignments, a few do local structural alignments. These include FOLDALIGN and LocaRNA, which conduct pairwise local structural alignments, as well as CMfinder [82].

## RNA Structure-Based ncRNA *In Silico* Screens

**Figure 11:**
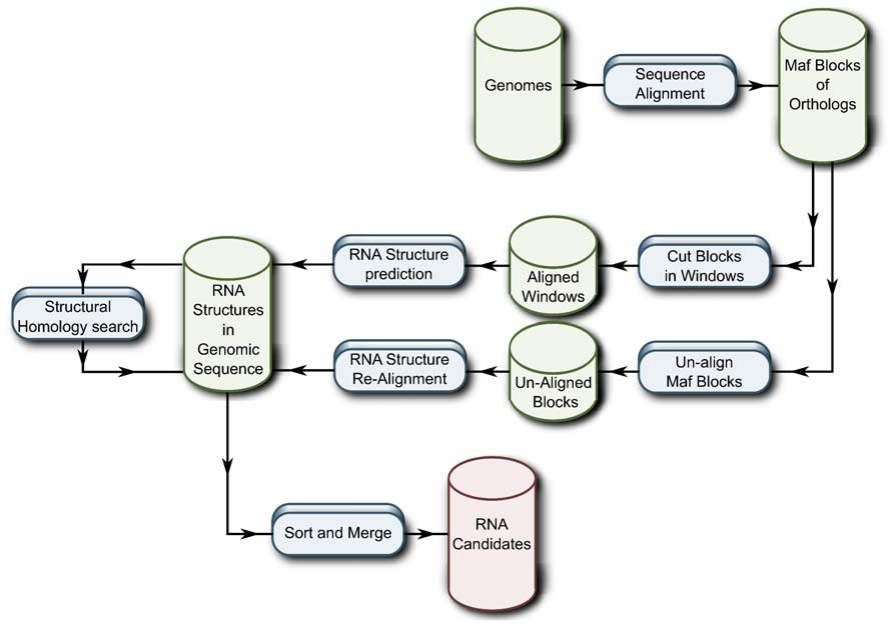
The basic flow of strategies for *de novo* prediction of RNA structures in genomic sequences. Given the strategy of applying multiple organism sequences, orthologs are already obtained. For the homology search using the obtained *de novo* candidates, these can be compared in syntenic regions as for obtained homology candidates. (Figure courtesy of Christian Anthon.)

Here, we describe the basic principles applied for the search of structured RNAs in genomic sequence and we refer to [42] for a detailed overview and discussion of the outcome. There are two main directions that have been applied for the *de novo* search for RNA structure, which is, as indicated above, a trade off between computational resources and the ability to explore the size of the search space. The two directions are, one that employs sequence-based alignments and one that also exploits synteny/orthology, but allows for structural (re-)alignment of the sequences. This is also sketched in Figure 11.

### Screens on Sequence-Based Alignments

These screens are typically carried out by using a sliding window, that is, a pre-defined window of some size is moved along a set of multiple aligned genomes (typically MAF [Multiple Alignment Format] blocks from the UCSC browser [83]). The alignment is based on sequence similarity and the window slides a number of nucleotides (e.g., half or quarter of the window size) in each step. In each window a consensus structure prediction is performed. By the end of the screen, various types of post processing are carried out, such as ranking the findings, estimating a false positive rate, determining strand specificity, and finding overlapping regions.

A potential drawback of the procedure is that results depend not only on the quality of the input alignments, but also on the windowing procedure. Windows should be large enough to fully cover ncRNAs (or at least a complete substructure), but should not be much larger than the smallest ncRNAs one wants to detect. A window size of, e.g., 120 nt, as has been used in RNAz screens (see below), is large enough to ensure that almost all miRNAs precursors will be detected. However, for maximum sensitivity, it can make sense to repeat screens using different window sizes.

An early reasonably successful attempt to predict structured RNAs from sequence alignments was qrna [84], which employed three different models of sequence evolution: a pair of HMMs describes the null model of sequences evolving without position dependent constraints, a second HMM that produces pairs of codons and models the evolution of protein coding sequences, and finally a pair of SCFGs is responsible for determining the evolution of sequence pairs with a common secondary structure. qrna computes the likelihood of the input alignment for each model, and identifies the model that yields the highest likelihood for the input alignment. qrna was successfully used to predict ncRNAs candidates in *E. coli* and *S. cerevisiae* [85], [86], some of which were verified experimentally. A limitation of qrna is that it only works on pairwise alignments. With the more recent method, Evofold [87] tries to extend the qrna approach of model comparison to multiple alignments. It adopts the pfold approach of modelling the joint probability of consensus structure and alignment by combining a phylogenetic model (substitution process along the branches of a tree) with a simple SCFG to compute the *a priori* probability of a structure.

In contrast to the SCFG-based approaches, the AlifoldZ and RNAz programs are based on energy-directed folding. In [65] it was shown that (in contrast to single-sequence folding) the joint folding energy of real ncRNAs can be distinguished from the folding energies of randomized alignments. A natural measure to assess whether an RNA is unusually stable is to compute a *z*-score over folding energies 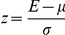 where 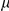 and 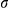 are the mean and standard deviation of randomized sequences obtained by shuffling. The idea in AlifoldZ is simply to compute the *z*-score using the energies of consensus structures as returned by RNAalifold. This is straightforward except that it requires a method to randomize alignments. Simply shuffling columns would result in alignments with unusual gap and conservation patterns (e.g., many short gaps instead of a few longer gaps). AlifoldZ therefore uses a conservative shuffling where only columns that display the same gap pattern and similar conservation can be swapped.

**Figure 12:**
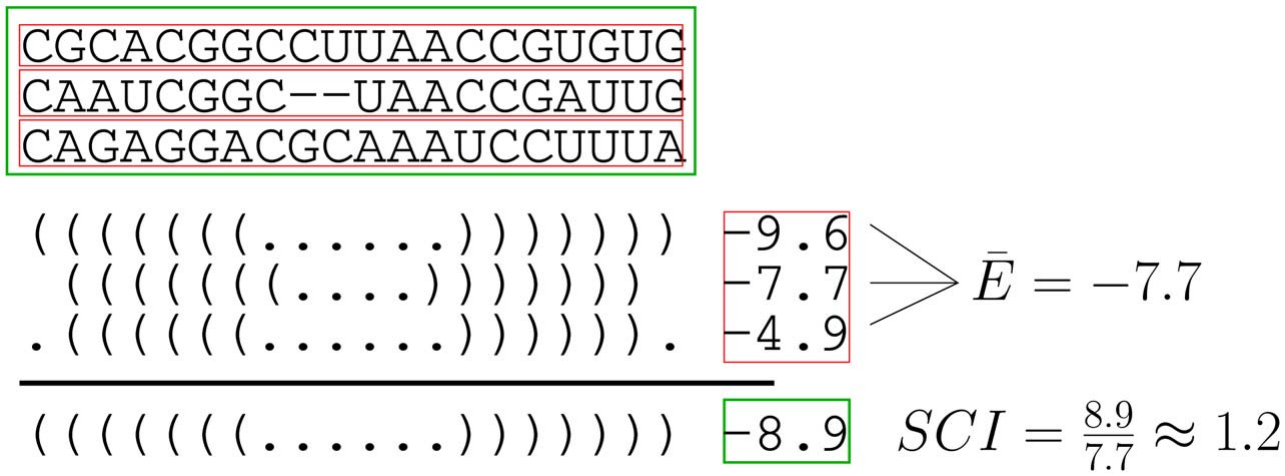
Computation of the SCI from a multiple alignment.

The shuffling procedure, however, results in a somewhat slow procedure. RNAz [88] therefore aims to avoid shuffling altogether. It uses energy *z*-scores for single sequences only and combines it with a separate measure of structure conservation. Importantly, the *z*-scores for single sequences can be estimated, as it turns out that the mean energy 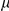 and standard deviation 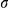 are simply functions of the sequence length and composition. RNAz therefore uses a support vector machine (SVM) (for a tutorial, see e.g., [89]) to train regression models for 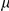 and 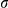, which allows computation of *z*-scores with only a single call to the folding algorithm. The latest version of RNAz [90] improves detection accuracy by using a regression model based on di-nucleotide content rather than nucleotide frequencies. To quantify structural conservation, RNAz uses a *structure conservation index* (SCI), defined as the ratio of the energy returned from consensus structure prediction 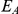 divided by the average folding energy of the individual sequences 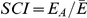, see Figure 12. Finally, a SVM takes the *z*-score and SCI as input and classifies the alignment (of the given window) as containing a significant RNA structure or not.

The Sankoff-based method Dynalign was applied in a screening approach using a fixed size window, but allowing for realignment (by Dynalign) and training of an SVM on such alignments. For low sequence similarity candidates (with identity less than 50%), it (not surprisingly) performs better than RNAz [91]. Subsequently, Dynalign has been optimized to lower its computational resources by employing an HMM for pre-processing the input and applying the HHM-based alignment as a constraint [74].

### Local Searches

A local search for RNA structure deviates from that of sequence-based alignments in two main ways. Firstly, even though the alignment is used to indicate orthology or synteny, the alignment itself is ignored and the combined sequence structure approach is applied to the sequences. Secondly, the approach is not bound by any window, so does not suffer from limitations such as adding too much flanking region and/or partial overlap to a real RNA structure, both of which can result in erroneous detection of RNA structures. In contrast, the local search approaches do not suffer from these limitations, but come with a set of their own to lower the computational overhead and make the methods practical. These limitations include a limited motif size, typically 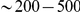 nt, though this might change in the future.

In the Sankoff-based approach FOLDALIGN, constraints other than those mentioned above made genome-wide screens possible. Two corresponding genomic sequences of lengths 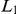 and 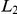 were screened, but since the motif size was limited to size 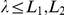, it was only necessary to store a 4D matrix constrained by 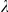 (typically 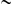
200 nt) rather than the full (large) sequence lengths. Essentially, the dynamical programming matrix slides along the two genomes and for each position throws away elements corresponding to positions no longer included by the motif range while adding new ones. To screen (genomic) sequences, one of the sequences is chopped into pieces of size 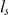, where a default value is 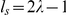 and where two consecutive pieces overlap 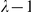 nucleotides. Without employing pruning, this doubles the running speed as compared to storing the entire 4D programming matrix in memory. This approach was applied to screen corresponding but unaligned sequences between human and mouse [92].

**Figure 13:**
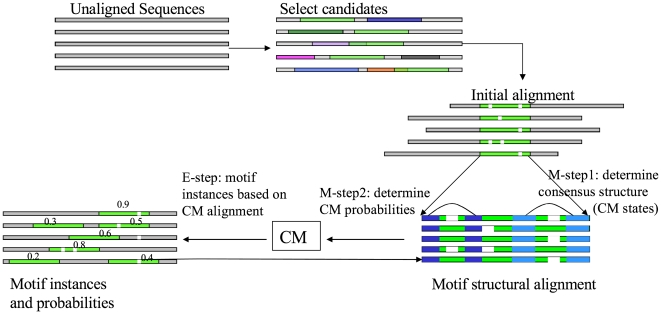
Searching unaligned sequences using CMfinder. After construction of an initial alignment (based on energy folded seeds), a covariance model is constructed and used to make further searches. Additional findings are incorporated into the model and novel searches are made until convergence was reached. (The figure was kindly provided by Zizhen Yao.)

While the current local alignment version of FOLDALIGN is limited to two sequences, it is also of interest to conduct a screen involving multiple sequences. The program CMfinder [82] searches a set of unaligned sequences using seed structures found from energy folding. It aims exactly to do what is outlined in Figure 1. The principle is summarized in Figure 13 and holds significant overlap to the early SCFGs [70]. The candidates are used to construct an initial alignment from which a covariance model is constructed and used to make further searches. Additional findings are incorporated into the model and a new search is made until convergence is reached. As in the work of Eddy and Durbin, an expectation maximization (EM) algorithm was employed to find the optimal local structure. CMfinder was also recently applied to screen for ncRNAs in prokaryotes [93], [94] and has been a main tool in riboswitch discovery, e.g., [94]. An additional strength is that if some of the sequences do not contain the RNA structure, they will simply be ignored, whereas the sequence alignment–based methods discussed above try to predict an RNA structure in all sequences.

An overview of the methods applied in *in silico* screens along with a short description of what they have been applied on can be found in [42].

### False Discovery Rates

A main issue that comes with all the methods for *de novo* RNA structure searches is they have high false positive rates, around 50% [42]. Furthermore, a comparison of the ENCODE regions [95] that comprise one percent of the human genome show little overlap between RNAz, Evofold, and CMfinder. Even though the methods work in quite different ways, they all aim to fulfill the same task. This clearly shows that the area still needs to mature. A future direction is to improve the background model for the screens, e.g., by using di-nucleotide shuffling [90]. A major challenge lies in providing good background models for shuffling multiple alignments. Recent advances in that area include methods like SISSIz [96] and Multiperm [97].

The Multiperm program shuffles the multiple alignments, while preserving gap and local patterns of conservation, while also preserving the approximate di-nucleotide frequencies, which is a main concern. The SISSIz program simulates (using a phylogenetic substitution model) a multiple alignment with a given dinucleotide content and does preserve, on average, local conservation patterns and gap structure. To our knowledge, the two programs have not been systematically benchmarked, but in our experience they are of approximately the same quality (unpublished observations).

### Performance Evaluation

Evaluating the performance of both RNA structure prediction and RNA gene finding is a subtle task. In both cases, a comparison to known (blinded to the experiment) data is required. RNA structure prediction is typically evaluated by comparison to curated structure data, e.g., [61]. From the number of (in)correctly predicted base pairs one computes accuracy measures, such as the positive predictive value (PPV) [98] and specificity, or Matthews correlation coefficient [99]. The latter is for RNA structure prediction well approximated by the geometric mean of the sensitivity (SEN) and PPV [100]. Note that the SCI measure is *not* suitable for performance evaluation, since it does not compare predictions to a blind dataset. SCI is a measure of divergence of the structures in the multiple alignment, and a high SCI does not necessarily imply correct performance, but merely states that the consensus structure is in good agreement with the structure of the individual sequences. Still, the entire structure prediction can be wrong.

For RNA gene finding, the genomic locations of predicted structures are compared to the locations of known RNAs (in blind dataset). Overlap of prediction and known gene (by some threshold) are used to state that a known RNA gene has been correctly predicted, see e.g., [81]. A major problem, however, is to measure the false positives, because a prediction in a given genomic location might indicate a so far unannotated ncRNA gene. What can be measured, however, is how many of the known ncRNA genes are missed in some benchmark dataset.

## Discussion

Approaches for *de novo* and *in silico* searching for structured RNAs is a highly difficult task that exceeds “regular” finding of protein coding genes in complexity due to the lack of regular patterns (such as codon bias). Algorithms have to take long-range interactions (secondary structure) into account, and typically work in a comparative manner requiring several homologous sequences.

The current algorithmic approaches using sequence-based alignments are much faster than using structural alignments [42]; however, structural alignments can take regions with weak sequence conservation into account more accurately. An observation from the CMfinder screen on the ENCODE regions was that the CMfinder alignment was similar to the original alignment for MAF blocks with high sequence similarity, but showed significant rearrangement for low similarity blocks [101].

A major challenge is the quality of currently available genomic MAF alignments. Especially if the number of species is large, alignment blocks are quite short such that an ncRNA may be broken up into multiple blocks. In this case one can try to extend or merge MAF blocks without losing too many species. Often, MAF blocks appear to be broken by gaps in one organism (unpublished observations).

When screening for RNA structures in genomic sequence, the respective methods optimizes a scoring function and within that function seeks an optimal structure. However, the structure predicted might well be suboptimal for a number of reasons. These include inaccuracies of the energy model, kinetic folding effects, as well as neglecting tertiary structure. Adding covariance information can dramatically improve the quality of structure prediction, but is dependent on the quality of the alignment. This is an issue in particular due to the limited quality of genome-wide alignments.

As the number of species grows, alignment blocks tend to become smaller. This imposes length constraints on the length of ncRNAs that can be detected, and may in the future require more sophisticated pre-processing of input alignments. In addition, the different search strategies have their own constraints on the length of their motifs (due to fixed window size, computational complexity, etc.) and thus have the same issues as for limited size MAF blocks. Currently, genomic screens typically result in a number of overlapping predictions, and the entire region is then merged into a candidate region for which there sometimes is not an entire structure prediction, e.g., [101], [102]. In fact, an open challenge is to make a good strand discriminator, as a prediction on one strand can imply an almost equally good prediction on the other strand. Some work has been initiated in this area [103].

Compensating base pairs are clearly important, even though systematic analyses to study the impact have not been carried out. As discussed in [42], the overlap between RNAz, EVOfold, and CMfinder on the ENCODE regions [95] was poor, and a main difference was that a substantial amount of CMfinder candidates had more and more of the MAF blocks re-aligned as sequence similarity dropped, suggesting that compensating base changes are important in lesser regions. In a study of known RNAs from Rfam, it was concluded the that MULTIz alignments were relatively accurate, but with room for better alignments in a number of regions [104]. Thus, a factor contributing to the lack of detection of novel RNAs could be the ability to include compensating changes into the alignments.

Suboptimal structures have not yet been taken systematically into account in ncRNA gene finding methods. However, at least in some cases, they might be essential for the detection of functional RNAs. Riboswitches, for example, are known to change conformation, and it is therefore expected that such types of information can add value to a genomic screen in general. To our knowledge, there have not been any systematic studies to compare predicted RNA structures from *in silico* screens with experimental data.

Simultaneously with the potential for RNA structure in the genome, a number of recent studies have shown the existence of long non-coding RNAs (lncRNAs), which are long transcripts. Presumably, these lncRNAs are largely unstructured [105]. However, recently, one of these lncRNAs was shown to have an enhancer-like function [106] that was coupled to the presence of short RNA structures in the lincRNA. These lncRNAs have been revealed to cover a variety functions [107], including epigenetic gene silencing [108], antisense regulation [109], and possibly chromatin organization, to promote long-range gene activation [110], to mention just a couple of examples. Over time there have been some attempts to distinguish coding from non-coding sequence on transcript. For a recent approach (post the lncRNA awareness), see [111].

Whether all lincRNAs contain local structured domains remains an open question. In [101] a functional RNA structure (67 nt) was predicted within a 2.8-kb ncRNA expressed in the brain, and subsequent studies revealed that this ncRNA also has overlap to RNAz predictions. Scenarios like this add to the challenge of arriving at full-length and/or functional transcripts from the RNA structure predictions, and it appears that RNA structure predictions cannot stand alone and will need to be accompanied by other types of data and possibly follow-up experiments to assign functional information.

Recently, exciting experimental developments have opened the arena for high-throughput structure probing on a transcriptome scale [112], [113]. These methods promise to provide useful data that can complement the computational screens, but are still in their early phase, each with their own challenges. For example, none yet work *in vivo*. Other sources for probing data are also promising to provide information applicable to a transcriptome-wide scale [114]. Incorporating such data in folding algorithms, including those used for genomic screens, will therefore be highly relevant. Emerging work in that area has recently been initiated [115].
